# The Fourth Annual Report

**Published:** 1916-09-09

**Authors:** 


### KING EDWARD VII. WELSH NATIONAL MEMORIAL ASSOCIATION.

### The Fourth Annual Report.

In spite of the war the report presented to the annual meeting of the Board of Governors of the Welsh National Memorial Association for the prevention and abolition of consumption, held on July 29, 1916, shows that the work of the Association has gone steadily forward. Although ten out of the twenty-seven medical officers who are employed by the Association were away on military service, there were actually substantial increases in the numbers of patients examined, treated in sanatoria, and admitted to hospitals.

The funds of this Association are derived from the County and Borough Councils of Wales, the (Welsh) National Insurance Commission, the Treasury, and from sundry other sources, such as interest on investments and donations from individuals. It is worthy of mention that the latter source, including moneys paid and promised up to March 31 last and also the properties of the Association, amounts in the four years of the Association's existence to over £228,000. Several interesting and generous gifts are recorded for the year under review. Thus Mr. D. S. Davies, late High Sheriff of Denbighshire, has promised to defray the cost—estimated at £2,285—of the projected surgical block of the North Wales Sanatorium at Llangwyfan; he desires it to be regarded as a gift from his daughter, Miss Nestor Davies, in memory of her mother. Unfortunately the scheme has had to be suspended until after the war, owing to the Treasury regulations, although the plans had been drawn up and approved by the Welsh Insurance Commissioners. It should be added that Mr. Davies in the first instance presented the site for this sanatorium, so that when the new block is erected his name will be doubly associated with it as benefactor.

Another interesting gift was a sum of £1,000 presented jointly by the three Messrs. Griffiths, brothers, of Newport, to pay for the alteration and equipment of Cardigan House, Newport, with a view to its conversion to the purposes for which it was presented by Sir Garrod Thomas and members of his family.

The proposal to establish there a hospital for surgical tuberculosis in children has given rise to considerable criticism, as our columns have recorded, and it would be reassuring if the association could issue a memorandum on the alleged drawbacks of the site from an institutional point of view.

It is not alone the developments at the North Wales Sanatorium that have been hindered by the war and its results. The projected hospital at Pontypool Road, on land given to the Association by Mr. J. C. Hanbury, is stopped for the same reason. Contracts for six hospitals and sanatoria entered into before the Treasury restrictions became operative are, however, to proceed. But in connection with all of them labour difficulties have been very considerable. Nevertheless, the Council is able to report that the extensions at the Glan Ely Hospital, near Cardiff, and the hospital at Llangefni, have been completed and opened for the reception of patients. Also the sanatorium at Llangwyfan (the surgical block excepted, as already mentioned) was so far advanced at the end of March that it was hoped to have it opened for the reception of a hundred patients before the end of the summer. The sanatorium at Talgarth is completed as far as the main buildings are concerned, though there is a great deal still to be done on the inside. Unfortunately there have been difficulties here in connection with the water supply, which are not yet overcome. The hospital at Tregaron, which should have been finished months ago but for scarcity of labour, was so far advanced when the Council drew up their report that they hoped to have it opened before that report was presented to the annual meeting.

Apparently every local authority in Wales has "come in" under the National Memorial Association, except the County Councils of Breconshire and Pembrokeshire. The former of these two is standing out only over some technical question on the form of the agreement, and measures are being taken which are expected to solve the difficulty. Pembrokeshire, however, seems to be definitely hostile and recalcitrant; as a result of this attitude, the Memorial Association feels obliged to refuse treatment of all except insured persons in this county. Without expressing any opinion on the merits of the dispute, it is surely obvious to both parties that this quarrel is undignified and unpatriotic, as well as unfair to the inhabitants (not being under the Insurance Acts) of that county. In view of the adhesion of the other counties to the Association, it seems highly probable that the major part of the blame must be allotted to the County Council in question.

An important supplemental charter has been adopted by the Memorial Association, after approval by the Treasury and the Welsh Insurance Commissioners. Its object is to effect an administrative change in the Association—namely, to alter the statutes which make the secretary the chief administrative officer, thus paving the way for a reorganisation which four years' experience of the work has shown to be necessary in the medical as well as the administrative departments. Medical men closely connected with the Association have expressed the opinion that the tuberculosis campaign is retarded by an excessive amount of lay control, and it would appear that the supplemental charter is a preliminary to certain changes directed to meet this criticism. For some time the medical director, Dr. Marcus Paterson, has been stationed at Alltymynydd Sanatorium at Llanybyther, which is too remote from Cardiff as the centre for effective medical control to be exercised. The Association now proposes to define clearly the duties of Mr. D. W. Evans, the general director, and Mr Gwilym Hughes, the secretary.

